# A110 INVOLVING STAKEHOLDERS TO DEVELOP BREASTFEEDING EDUCATIONAL RESOURCES FOR PATIENTS WITH INFLAMMATORY DISEASE

**DOI:** 10.1093/jcag/gwad061.110

**Published:** 2024-02-14

**Authors:** S Rho, J Ferenbok, E Marcon, P Habashi, K Berga, L Bradbury, V Huang

**Affiliations:** Sinai Health, Toronto, ON, Canada; University of Toronto, Toronto, ON, Canada; University of Toronto, Toronto, ON, Canada; Sinai Health, Toronto, ON, Canada; St. Lawrence College, Ottawa, ON, Canada; The Hospital for Sick Children, Toronto, ON, Canada; Sinai Health, Toronto, ON, Canada

## Abstract

**Background:**

Inflammatory Bowel Disease (IBD) are incurable chronic inflammatory disease of the digestive tract that is often treated with immunosuppressive medications. Common IBD symptoms are frequent bowel movements that may be accompanied by blood, constipation, diarrhea, abdominal cramps and pain, fatigue, unintentional weight loss, and many more. Patients who are or are considering breastfeeding often face concerns and decisions about IBD medications and potential effects on breastfeeding and their breastfed infant, or they may have trouble with breastfeeding due to IBD symptoms. Despite the evident benefits of breastfeeding for infants, the rate of long-term breastfeeding amongst women with IBD is low at 55.2% at 6 months. A preliminary environmental scan revealed that there is a lack of informative educational resources for the population.

**Aims:**

To develop a patient-centric educational resource that provides information and assurance on breastfeeding for a person living with IBD.

**Methods:**

The study involved 4 stages: (1) phase 1 interview to collect perspectives and expertise, (2) prototype development, (3) phase 2 interview to obtain feedback on the prototype, and (4) prototype refinement. Participants included two groups: healthcare providers and IBD patients who have breastfed or are currently breastfeeding. All interviews were conducted remotely. Interview transcripts were coded to analyze the data.

**Results:**

In total of 12 participants (6 healthcare providers and 6 patients) were recruited and completed the study. Through thematic analysis, three main themes were identified: 1) breastfeeding concerns that patients have prior to breastfeeding; 2) breastfeeding issues that patients experience while breastfeeding; 3) education format and content. These themes aided in the development and refinement of the prototype, which included graphics, easily digestible information, references, and short videos. The prototype is a webpage residing in a preconception and pregnancy in IBD website.

**Conclusions:**

By involving the stakeholders, the intervention reflected the needs of patients and the expertise of healthcare providers. The main concern of IBD patients regarding breastfeeding was the IBD medication transfer via breastmilk and safety for breastfed infants. Interestingly, patients preferred to obtain information through short videos rather than the traditional counseling method that healthcare providers offer in clinics. These are included in the intervention. This study method can be applicable to other fields, to ensure that the needs of patients are well represented in patient education resources.

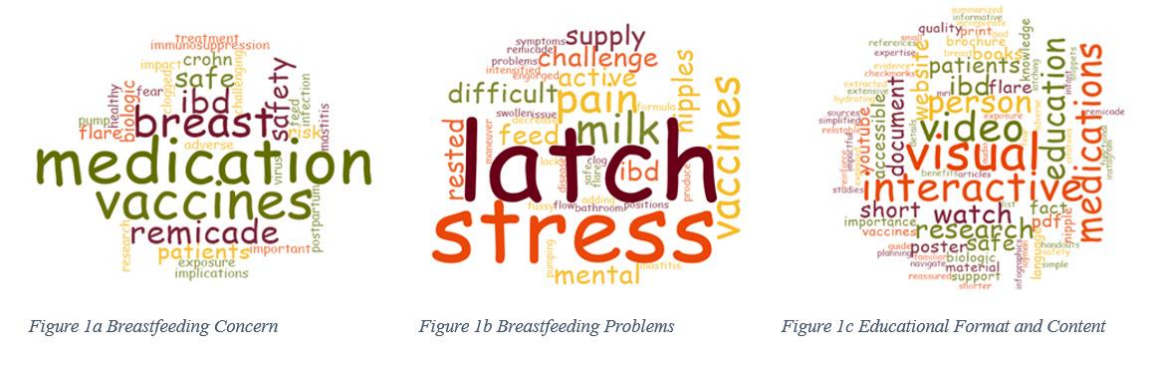

**Funding Agencies:**

University of Toronto

